# CB2R Activation Regulates TFEB-Mediated Autophagy and Affects Lipid Metabolism and Inflammation of Astrocytes in POCD

**DOI:** 10.3389/fimmu.2022.836494

**Published:** 2022-03-22

**Authors:** Lieliang Zhang, Xifeng Wang, Wen Yu, Jun Ying, Pu Fang, Qingcui Zheng, Xiaojin Feng, Jialing Hu, Fan Xiao, Shoulin Chen, Gen Wei, Yue Lin, Xing Liu, Danying Yang, Yang Fang, Guohai Xu, Fuzhou Hua

**Affiliations:** ^1^ Department of Anesthesiology, The Second Affiliated Hospital of Nanchang University, Nanchang, China; ^2^ Key Laboratory of Anesthesiology of Jiangxi Province, Nanchang, China; ^3^ Department of Anesthesiology, The First Affiliated Hospital of Nanchang University, Nanchang, China; ^4^ Department of Neurology, The First Affiliated Hospital of Nanchang University, Nanchang, China

**Keywords:** cannabinoid type 2 receptor, astrocytes, autophagy, mitochondrial damage, lipid accumulation, inflammation, postoperative cognitive dysfunction

## Abstract

Evidence suggests that the accumulation of lipid drots (LDs) accelerates damage to mitochondria and increases the release of inflammatory factors. These have been implicated as a mechanism underlying neurodegenerative diseases or tumors and aging-related diseases such as postoperative cognitive dysfunction (POCD), nevertheless, accumulation of lipid droplets has not been extensively studied in the central nervous system (CNS). Here, we found that after surgery, there was activation of astrocytes and lipid accumulation in the hippocampus. However, cannabinoid receptor type II (CB2R) activation significantly reduced lipid accumulation in astrocytes and change the expression of genes related to lipid metabolism. CB2R reduces the release of the inflammatory factors interleukin-1 beta (IL-1β) and interleukin 6 (IL-6) in peripheral serum and simultaneously improves cognitive ability in mice with POCD. Further research on mechanisms indicates that CB2R activation promotes the nuclear entry of the bHLH-leucine zipper transcription factor, the transcription factor EB (TFEB), and which is a master transcription factor of the autophagy–lysosomal pathway, also reduces TFEB-S211 phosphorylation. When CB2R promotes TFEB into the nucleus, TFEB binds at two sites within promoter region of PGC1α, promoting PGC1α transcription and accelerating downstream lipid metabolism. The aforementioned process leads to autophagy activation and decreases cellular lipid content. This study uncovers a new mechanism allowing CB2R to regulate lipid metabolism and inflammation in POCD.

## Introduction

Postoperative cognitive dysfunction (POCD), which seriously affects the quality of life of elderly postoperative patients, needs to be addressed urgently in current postoperative complications. Despite the high morbidity of POCD, no treatments have been established. Thus, novel therapeutic strategies to treat brain injuries secondary to POCD, such as neuronal death, glial cell-mediated neuroinflammation, and astrocyte metabolism disorders, are necessary to improve the mortality and disability rates of patients with POCD (1, 2).

Recent evidence suggests that astrocyte-mediated lipid accumulation and inflammation are important factors in the development of neurodegenerative diseases (3, 4). Lipid drots (LDs), usually stored as neutral lipids in the cell, are essential for lipid metabolism and energy homeostasis. LD dysfunction has been associated with the occurrence and development of various diseases. Increasingly accumulating evidence indicates a considerably broader role of LDs in disease occurrence and progression than previously recognized. The accumulation of lipids on astrocytes has been associated with aging-related diseases. For instance, lipid dysregulation and energy deficits in astrocytes increased the risk of AD (5). In the latter study, astrocytes were particularly sensitive to lipid toxicity and triggered downstream inflammation. Failure to promptly clear the accumulation of LDs leads to accelerated LD peroxidation, causing neuroinflammation and nerve damage. Excessive LD content may trigger nonoxidative metabolic pathways and cellular toxic reactions. Recent studies have also shown that in LD-deficient cells, fatty acid (FA) is converted to acylcarnitine, leading to mitochondrial dysfunction and reactive oxygen species (ROS) production (6). These processes may exert specific and far-reaching effects on astrocytes. Dysfunctional mitochondria damage the ability of FA consumption, and the production of mitochondrial reactive oxygen species causes further membrane lipid peroxidation.

Cannabinoid receptor type II(CB2R)belongs to the G protein-coupled family of receptors (GPCRs), unlike the cannabinoid receptor type I (CB1R) CB1R, which is located mainly in the CNS and whose activation can exert harmful cognitive effects (7). Although expressed predominantly in the peripheral nervous system, CB2R is also expressed in the immune system (8). Recent research has found that CB2R is substantially involved in CNS activities. Specifically, microdialysis and voltammetry studies revealed insights into the effects of CB2R signaling on neurotransmitter release. The CB2R agonist JWH133 dose-dependently reduced dopamine (DA) neuron firing in the ventral tegmental area (VTA) in wild-type mice. This effect was blocked by the co-administration of AM630 and was not present in CB2R-KO mice (9). In another study, the systemic administration of AM630 could impair the consolidation of aversion memory, whereas the CB2R agonist JWH133 enhanced the consolidation of aversion memory by inhibitory avoidance testing (10). In addition, fear increased the gene expression of CB2R but not of CB1R in the hippocampus, which is related to anxiety-related behaviors, such as freezing and escape latency. CB2R modulated the neuroinflammatory and cognitive impairment in a mouse model of orthopedic surgery, and the activation of CB2R effectively alleviated hippocampus-dependent memory loss in mice in the early postoperative stage (11). In POCD, the mechanism allowing CB2R action on astrocytes in the regulation of cognitive function remains unclear.

Transcription factor EB (TFEB) is a basic helix–loop–helix–leucine–zipper transcription factor, which is considered the main regulator of lysosomal biogenesis and autophagy, and mediates various physiological processes, including immune response, neurodegenerative diseases, and metabolic diseases (12–15). TFEB is usually phosphorylated by the kinase mTORC1 at S142 and/or S211, and is retained in the cytoplasm by binding to protein 14-3-3 (16, 17). However, under external stimuli such as starvation, dephosphorylated TFEB is transported to the nucleus and combines with the coordinated lysosomal expression and regulated coordinated lysosomal expression and regulation (CLEAR) element. TFEB also regulates the genes involved in lipid metabolism *via* PPARGC1A PPARG coactivator 1 alpha (PGC1α) (17, 18).

In the present study, CB2R activation promoted the nuclear entry of TFEB and simultaneously reduced TFEB-S211 phosphorylation. TFEB was bound to the promoter region of PGC1α, promoting PGC1α transcription and accelerating downstream lipid metabolism, reducing the expression of inflammatory factors IL-1β and IL-6 in cells. In this process, LDs were encapsulated in autophagosomes and decomposed into free FAs in autophagolysosomes and then entered mitochondria to complete oxidation. Moreover, lipid overload led to blockage of autophagy.

## Materials and Methods

### Animals

12-month-old male C57BL/6J mice, obtained from Medical Animal Experiment Center of Nanchang University, were bred and maintained in the Animal Resource Centre of the Faculty of Nanchang University. Mice were given free access to food and water in a controlled environment with an ambient temperature of 22°C ± 2°C, a 12:12–h light/dark cycle and a humidity of 60%. All animal procedures were conducted in strict accordance with the Guide for the Care and Use of Laboratory Animals (National Institutes of Health, the United States) and the guideline of the Institutional Animal Care and Use Committee of Nanchang University.

### Surgical Model

After one-week acclimatization, mice were subjected to an intramedullary fixation surgery for tibial fracture under isoflurane anesthesia (19). Briefly, mice were fasted for 12 h before surgery, then anesthetized with Briefly, the mice were anesthetized with 1.8% isoflurane and oxygen at 2 L/min in the small animal anesthesia machine (R500, RWD Life Science). A skin incision was made below the knee to expose the tibia, and a 0.3 mm pin was inserted into its medullary cavity for intramedullary fixation. Next, the bone was fractured at the midpoint with a surgical scissor. Lastly, the wound was sutured after necessary debridement. 2% lidocaine solution was applied locally before the incision, and 1% tetracaine hydrochloride mucilage was applied to the wound twice daily to treat the pain.

### Intracerebroventricular (ICV) Injection

We refered to the methods in the literature and apply them in our experiments (20).After anesthetizing the mice with sodium pentobarbital, mice were placed on the stereotaxic apparatus, drill a 2mm small hole was drilled by a dental drill, and a 26-G stainless steel tube implanted into the right ventricle of the mouse. The guide sleeve was fixed by dental cement, fixed by stainless steel screws fixed on the skull, and sealed with stainless steel wire to prevent blockage. Injections into lateral ventricles were performed using the following coordinates from Bregma: 0.8 mm posterior, 0.2 mm lateral, and 2.4 mm ventral. JWH133 (3μg) or AM630 (3μg) were infused intoventricle at a rate of 1 μl/min using a microinjector (World Precision Instruments) fitted with a 30 gauge needle attached to a 10 μl gas-tight syringe (Hamilton, 7634-01). The needle was left in place for 2 min post injection before slowly withdrawing.

### Immunofluorescence Staining and BODIPY Staining

Hippocampus tissue immunofluorescence staining was performed as previous described (21). Frozen 30-μm-thick brain slices were washed three times with phosphate-buffered saline (PBS) and then incubated with 0.3% Triton X-100 in PBS supplemented with 5% bovine serum albumin (BSA) for 1 h at room temperature. Then, brain slices were incubated with specific primary antibody (Mouse anti-GFAP antibody, 1:500, MAB360, Millipore;Rabbit anti-IL-1β antibody,1:500,31202,Cell Signaling Technology) at 4°C for overnight. The next day after incubation with the fluorescent secondary antibody (1:1000, A21422/A-11008, Invitrogen) at room temperature, the nucleus was stained with Hoechst (1:1000, B2261, Sigma-Aldrich). For BODIPY staining, incubating the brain slices after diluting BODIPY dye (D3861, Invitrogen) with PBS, and finally washing 3 times with PBS.The brain slices were placed on slides and observed under a fluorescence microscope (Olympus, Tokyo, Japan).

### RNA Isolation and Quantitative Real Time PCR (RT-PCR)

Briefly, total RNA from hippocampus tissues or U87 cells were extracted using Trizol Reagent and then reversely transcribed into cDNA using PrimeScript™ RT Master Mix. Real-time qPCR was carried out using SYBR Green Master Mix (Applied Biosystems) in a StepOnePlus instrument (Applied Biosystems). Relative gene expression was analyzed using the comparative 2−^ΔΔCt^ method (22). glyceraldehyde-3-phosphate dehydrogenase (GAPDH) was used as a normalization control. The primers were purchased and validated from Generay (Shanghai, China) and the sequences used are as follows:

**Table:** 

	Forward	Reverse
Crat	5’-GCTGCCAGAACCGTGGTAAA-3’	5’-CCTTGAGGTAATAGTCCAGGGA-3’
Cyp4a12a	5’-CCTCTAATGGCTGCAAGGCTA-3’	5’-CCAGGTGATAGAAGTCCCATCT-3’
Acacb	5’-CGCTCACCAACAGTAAGGTGG-3’	5’-GCTTGGCAGGGAGTTCCTC-3’
Gpx4	5’-TACGGACCCATGGAGGAG-3’	5’-CCACACACTTGTGGAGCTAGAA-3’
Sod	5’-CGGAGTCAACGGATTTGGTCGTAT-3’	5’-AGCCTTCTCCATGGTGGTGAAGAC-3’
Cat	5’-CGGAGTCAACGGATTTGGTCGTAT-3’	5’-AGCCTTCTCCATGGTGGTGAAGAC-3’
Nox1	5’-CGGAGTCAACGGATTTGGTCGTAT-3’	5’-AGCCTTCTCCATGGTGGTGAAGAC-3’
Nox2	5’-CGGAGTCAACGGATTTGGTCGTAT-3’	5’-AGCCTTCTCCATGGTGGTGAAGAC-3’
Nox4	5’-CGGAGTCAACGGATTTGGTCGTAT-3’	5’-AGCCTTCTCCATGGTGGTGAAGAC-3’
GAPDH(Mouse)	5’-AATGGATTTGGACGCATTGGT-3’	5’-TTTGCACTGGTACGTGTTGAT-3’
Sqstm1	5’-GCACCCCAATGTGATCTGC-3’	5’-CGCTACACAAGTCGTAGTCTGG-3’
Lamp1	5’-TCTCAGTGAACTACGACACCA-3’	5’-AGTGTATGTCCTCTTCCAAAAGC-3’
Ctsd	5’-TGCTCAAGAACTACATGGACGC-3’	5’-CGAAGACGACTGTGAAGCACT-3’
Ctsb	5’-GAGCTGGTCAACTATGTCAACA-3’	5’-GCTCATGTCCACGTTGTAGAAGT-3’
Atp6v1d	5’-AGCACAGACAGGTCGAAACC-3’	5’-TTCTCTCATCACTTCGCCCAT-3’
Atp6v1h	5’-CAGAAGTTCGTGCAAACAAAGTC-3’	5’-TCAGGGCTTCGTTTCATTTC>AA-3’
PGC1α	5’-TCTGAGTCTGTATGGAGTGACAT-3’	5’-CCAAGTCGTTCACATCTAGTTCA-3’
IL-1β	5’-ATGATGGCTTATTACAGTGGCAA-3’	5’-GTCGGAGATTCGTAGCTGGA-3’
IL-6	5’-ACTCACCTCTTCAGAACGAATTG-3’	5’-CCATCTTTGGAAGGTTCAGGTTG-3’
GAPDH(Human)	5’-TGTGGGCATCAATGGATTTGG-3’	5’-ACACCATGTATTCCGGGTCAAT-3’

### ELISA

Serum samples were collected from eyeball blood in anesthetized mice, and the concentration of IL-1β(R&D,MHSLB00),IL-6 (R&D, M6000B) were measured by mouse IL-1β and IL-6 ELISA Kits according to the manufacturer’s instructions.

### Behavioral Assessment

#### Y-Maze

Y-maze was performed referring to literature concerned (23) and used the CLEVER-SYM system to record the mouse movement route. The Y-maze is composed of a 34-cm starting arm, a 10-cm novel arm, and 10-cm other arms. Mice were placed in the test room 24 hours before the experiment to accommodate the environment. At the beginning of the test, the novel arm was blocked with a baffle and mice were placed from the starting arm. The mice were free to explore the starting arm and the other arms for 5 minutes before starting to record their movement trajectory. One hour later, the mice were placed in the starting arm, the new arm was opened for a 5-minute exploration, and the movement traces were recorded. The function of short-term memory in mice was measured by dividing the time spent exploring the novel arm by the total time.

#### Novel Object Recognition

Novel object recognition assay is a relatively sensitive procedure for evaluating cognition-enhancing activities in mice. First, two same objects were placed in an autonomously moving box (40 × 27 × 20 cm^3^) near the sides. The mice were then gently placed in the box and explored freely for 5 min, recording the time spent exploring each object (exploration was defined as sniffing or touching the object with the nose within 0-2 cm radius). After 5 min, one of the objects was replaced with a new one, the mice were allowed to continue exploring for 5 min, and the length of time the animals spent exploring the old and new objects was recorded. The mouse movement route was recorded by CLEVER-SYM system. New object recognition rate = new object recognition time/(new object recognition time + old object recognition time) * 100%.

### Injection of Adeno-Associated Viral Vectors (AAVs)

Mouse siRNA was transfected with packaging vectors (AAV9-GFAP-Null (CB2R)-bGHpolyA) to generate AAV. For microinjection, intraperitoneal pentobarbital sodium-anesthetized mice were placed in a stereotaxic apparatus. They were injected with the 1 μL AAV by glass electrode aiming in hippocampus (AP: −0.18 mm; ML: ± 0.1 mm; DV: −0.2 mm) at a rate of 0.2 μL/min. Then it needed additional 2-minute needle retention. POCD model was conducted 2 weeks after virus microinjection.

### Immunohistochemical Analysis

The hippocampus tissue slices were washed three times with PBS, followed by 3% H_2_O_2_, for 15 min to quench the endogenous peroxidase activity, and then incubated with 0.3% Triton X-100 in PBS supplemented with 5% BSA for 1 h at room temperature. The brain slices were incubated with the specific primary antibody (Mouse anti-GFAP antibody, 1:500, MAB360, Millipore) at 4°C overnight. After incubating the slices with the secondary antibody the next day, they were incubated with diaminobenzidine for 5 min. PBS was used to stop the diaminobenzidine color reaction. The brain slices were dehydrated in alcohol and xylene and observed under a stereomicroscope (Olympus). The proportion of positive area was calculated using ImageJ software.

### Nissl Staining

The brain sections (30μm) were hydrated in 95%, 85%, and 70% gradient of ethanol for 5 min each. After washing with distilled water three times for 5 min each, they were stained with 0.1% cresyl violet (Solarbio, G1430) for 10 min. Then they were washed with distilled water, dehydrated in gradient alcohol, cleared with xylene, and sealed with neutral gum. The images were taken with Olympus VS120 Virtual Slide Scanner.

### Golgi-Cox Staining

After the mice were decapitated under deep anesthesia, the brains were quickly removed and washed with distilled water. The hippocampus tissue was immersed in the A and B impregnation solution (#PK401, FD NeuroTechnologies, MD, USA) and stored at room temperature for 14 days in the dark. Next, the brain tissue was transferred to the C solution and stored at room temperature in the dark for 7 days. Finally, it was embedded with the OCT glue to make a brain slice of about 100–200 μm, which was pasted on a glass slide containing C solution, treated with gelatin, and stored in the dark. After dyeing with D and E solution, the slices were dehydrated in alcohol and xylene and observed under a stereomicroscope (Olympus). The pictures taken were analyzed with the NeuroJ plugin in ImageJ software.

### Cell Culture and Treatment

The U87 human GBM cell line and 293T cell line were purchased from the Shanghai Institute of Cell Biology (Shanghai, China) and cultured in DMEM (A4192101, Gibco) supplemented with 10% fetal bovine serum (FBS, F8687, Sigma-Aldrich) at 37℃ in a humidified 95% air and 5% CO^2^ atmosphere. LPS/JWH133/AM630 treatment group: U87 cells were pretreated with 1μM JWH133 and 1μM AM630 for 1h, then treated with 100ng/mL LPS for 6h.

### BODIPY581/591, JC-1, Mitochondrial Tracker Green Staining

The U87 cells were incubated with BODIPY 581/591 C11 (BDC11) (2.0 μM, Invitrogen, D3861) in PBS for 15 min at 37°C. For JC-1 staining, live cells were incubated with 10μM JC-1 (Invitrogen, T3168) in PBS for 30 min at 37 ^◦^C (Mitochondrial Tracker Green (Invitrogen, M7514) staining method is the same as JC-1). The cells needed to be washed twice in PBS and fixed in 4% PFA for 10 min before Hoechst staining. The mounted slides were allowed dry for 1 h and images were observed and photographs were captured by fluorescence microscopy (Olympus, Tokyo, Japan) and confocal microscope (Zeiss, Germany).

### Flow Cytometry Analysis of BODIPY, JC-1 Staining

After staining the cells as above, the cells were dissociated with 0.25% trypase then analyzed using a Guava easyCyte System 8 (Millipore 25801, CA, USA).

### Cell Transfection

siRNA targeting TFEB and PGC1α (Jima, Shanghai, China) were transfected in U87 cells using Lipofectamine RNAimax reagent (13778150, Invitrogen) in Opti-MEM medium (Gibco) according to the manufacturer’s instructions. siRNA duplexes used later, the transfection mixture was removed after 12h, and cells were further incubated with a normal medium for an additional 36 h before LPS stimulation. In overexpression experiments, the cells were transfected with TFEB plasmids using Lipofectamine 3000 (L3000-008, Invitrogen).

### Tandem Fluorescent-mRFP–GFP–MAP1LC3B–Adenovirus Transduction of U87

The U87 cells were transfected with a tandem fluorescent mRFP-GFP-MAP1LC3B adenovirus (HanBio) according to the manufacturer’s protocol. The green fluorescence of GFP and red fluorescence of RFP of the cells indicate the degree of obstruction and smoothness of the autophagic process. Six fields of view in each group of cells were selected for the assay. After the cells were processed, they were fixed in 4% PFA, rinsed with PBS, stained with Hoechst, and photographed. The ratio of green fluorescent spots to red fluorescent spots was calculated to reflect the process of autophagy.

### Cell Immunofluorescence Staining

The cell slides in the 24-well plate were washed three times with 0.1M PBS and fixed with 4% paraformaldehyde for 15 min. The paraformaldehyde was aspirated, washed with PBS, and blocked with PBS containing 5% BSA for 1 h. Then, the cell slide was incubated with the specific primary antibody (TFEB, 37785, Cell Signaling Technology) at 4°C overnight, and the fluorescent secondary antibody (Donkey anti-Rabbit IgG (H+L) Highly Cross-Adsorbed Secondary Antibody, Alexa Fluor 555, A-31572,ThermoFisher) was incubated for 1 h at room temperature the next day. After aspirating the secondary antibody and covering the cell slide with a glass slide, the cells were observed under the stereomicroscope (Olympus).

### Nuclear and Cytoplasmic Protein Extraction

The nuclear and cytoplasmic protein was extracted from U87 astrocytes using nuclear and cytoplasmic protein extraction kit (ThermoFisher,78833) according to the manufacturer’s instructions. Then the samples were analyzed by western blotting analysis.

### Plasmid Construction

GV143-TFEB, pcDNA3.1-TFEB plasmid were purchased from Genechem (Shanghai, China). GV143-TFEB was changed to GV143-S211A and GV143-S142A by site-specific amino acid mutations by using QuikChange II XL Site-Directed Mutagenesis Kit (Agilent, 200524). The above plasmids were verified by sequencing.

### Western Blotting Analysis

Cells were homogenized in pre-chilled RIPA lysis buffer (150 mM NaCl, 1 mM EDTA, 50 mM Tris, 1% Triton, 0.1% sodium dodecyl sulfate, and 0.5% deoxycholic acid) with protease inhibitors. The lysate was centrifuged at 16000 g for 15 min at 4°C to remove the precipitate. The total protein concentration derived was determined using the BCA protein assay kit (23225, Thermo). The 30-μg protein aliquot of each sample was separated using sodium dodecyl sulfate-polyacrylamide gel electrophoresis and was electrophoretically transferred onto PVDF membranes (IPVH00010, Millipore). The membranes were then blocked with 5% nonfat milk in Tris-buffered saline (20 mM Tris-HCl, 500 mM NaCl, pH 7.4) with 0.3% Tween 20 (Aladdin, T104863) for 1 h at room temperature. After blocking, PVDF membranes were incubated with various specific primary antibodies (TFEB:1:1000, 37785, Cell Signaling Technology; GAPDH:1:3000, 5174, Cell Signaling Technology; LaminB1:1:2000, 17416, Cell Signaling Technology) in TBST at 4°C overnight then washed and incubated in corresponding horseradish peroxidase (HRP) conjugated secondary antibodies (1:2000, 31460, Thermo Scientific) for 1 h at room temperature. Immunoreactive bands were visualized and detected by enhanced chemiluminescence (ECL) plus detection reagent (Pierce, Thermo Fisher Scientific, Rockford, IL) and analyzed using the ImageQuant™ LAS 4000 imaging system (GE Healthcare, Pittsburgh, PA, USA).

### Chromatin Immunoprecipitation (ChIP) Assay

Chromatin from U87 cells was fixed and immunoprecipitated using the ChIP assay kit (Cell Signaling Technology, 9005) according to the manufacturer’s instructions. The purified chromatin was immunoprecipitated using 2 mg of anti-TFEB. The presence of the selected DNA sequence was assessed by real-time PCR. ChIP signals were generated from 4 independent experiments followed by normalization to input signals and presented as mean ± SEM.

### Dual-Luciferase Reporter Assay

The firefly luciferase reporter vector pGL3 (Jima, Shanghai, China) and the constitutively active Renilla luciferase control plasmid pRL-TK (Jima, Shanghai, China) were used. The cells were washed with PBS and lysed with a RIPA lysis solution. The relative luciferase activity was measured at a wavelength of 410 nm using a plate reader (BD Biosciences) and normalized to Renilla luciferase activity (Promega, E1910). All procedures were carried out according to the manufacturer’s protocols.

### Statistical Analysis

All data were expressed as mean ± SEM and analyzed in a blind way using the Student t test, one-way analysis of variance (ANOVA), or two-way ANOVA followed by Tukey’s *post hoc* test.In animal experiments, n represents the number of samples per group. In cell experiments, n represents n times independent experiments. ^*^ *P* value <0.05 indicated a statistically significant difference.

### CB2R Activation Was Linked to Reductions in Lipid Accumulation and Inflammation in Astrocytes in Mice With POCD

**Figure 1:**
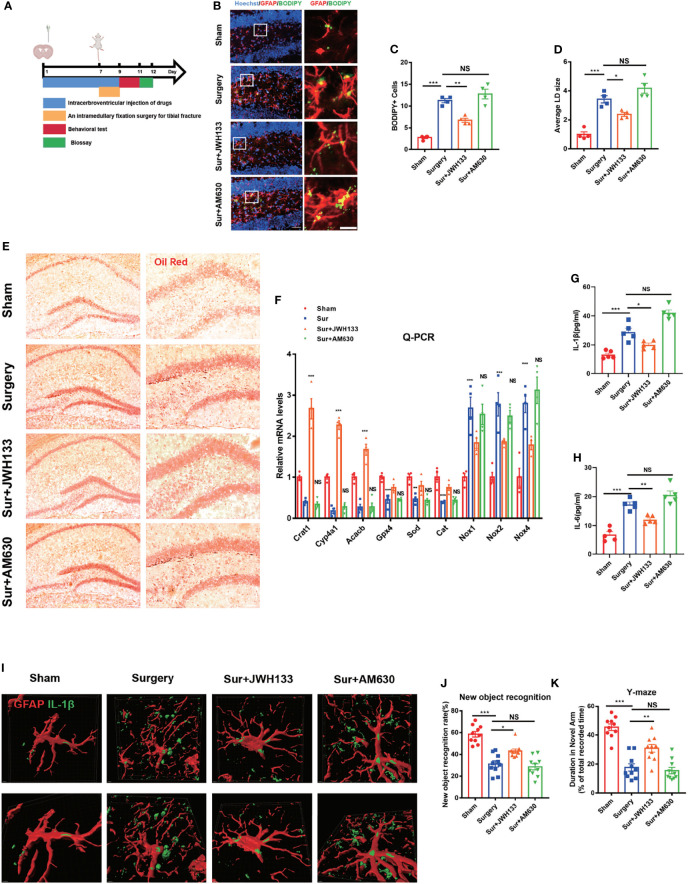
**(A)** Experimental protocol and timeline. Administration of JWH133 or AM630 *via* the lateral ventricle of the mice by using a stainless steel tube 7 d before surgery; behavioral and biological testing 2 d after surgery. **(B)** Immunofluorescence staining of GFAP and BODIPY staining in the hippocampal DG brain region. Statistical data on BODIPY+ cells, with the average LD size **(C, D)** (means±SEM, n=4, scale bars=40 and 3 μm). **(E)** Oil Red O LD staining in the hippocampus (n=4, scale bars=200 and 100 μm). **(F)** RT-qPCR results for the relative mRNA expression levels of Crat, Cyp4a12a, Acacb, Gpx4, Sod, Cat, and Nox1,2,4 in the hippocampus (means±SEM, n=4). **(G, H)** ELISA of IL-1β and IL-6 serum levels (n=5). **(I)** Co-labeling of GFAP and IL-1β in the hippocampus, as detected by immunofluorescence assay. Analysis of the fluorescence signal by using Imaris 9.0 to generate a 3D model (scale bars=2 μm). **(J, K)** New object recognition and Y-maze testing of mice in each group (means±SEM, n =10). NS means not significant, ^*^ *P* < 0.05, ^**^ *P* < 0.01, ^***^ *P* < 0.001 compared with the corresponding group, as determined by ANOVA.

Growing evidence suggests that lipid accumulation and inflammation in brain astrocytes are associated with an increase in neurodegenerative diseases, aging-related disorders, and brain damage, including but not limited to AD, PD, and traumatic brain injury (4, 24–26). Clinical evidence shows that the serum levels of lipids and inflammatory factors were highly elevated in aging patients undergoing surgery (27–29). Low-density lipoproteins can usually be degraded by autophagy, preventing excessive lipid accumulation that leads to neurological injury and apoptosis. To determine whether CB2R is involved in the accumulation of lipids in astrocytes, the CB2R agonist JWH133 and the CB2R inhibitor AM630 were injected into the bilateral ventricles, and a tibial fracture model was established (**Figure 1A**). Histological staining of glial fibrillary acidic protein (GFAP) with BODIPY, a dye that specifically labels neutral lipids and is commonly used to detect LDs, showed that the CB2R agonist significantly reduced the size and BODIPY^+^ cell numbers of LDs, relative to those of the surgery group **(** **Figures 1B–D** **)**. However, the aforementioned effects were not observed in the presence of AM630. To confirm these findings, we performed Oil Red O LD-specific staining in the hippocampus and observed LD deposition in the Surgery and Sur+AM630 groups **(** **Figure 1E** **)**. Literature reviews indicate that the lipid accumulated in astrocytes cannot be promptly metabolized and degraded; moreover, they trigger mitochondrial stress caused by lipid peroxidation, intensifying the damage by disease pathologies (30). Therefore, we further examined the lipid metabolism-associated genes carnitine acetyltransferase (Crat), cytochrome P450, family 4, subfamily a, polypeptide 12a (Cyp4a12a), and acetyl-coenzyme A carboxylase beta (Acacb). We found that CB2R agonists promoted the expression of lipid metabolism-related genes (Crat, Cyp4a12a, Acacb). Quantitative PCR analysis results also suggested a significant increase in essential antioxidant glutathione peroxidase 4 (Gpx4), catalase (Cat), and superoxide dismutase (Sod) mRNA expression in the agonist-treated group relative to those in the surgery group; meanwhile, the expression levels of the NADPH oxidase subunit Nox1,2,4 were decreased i, whereas the inhibitor treatment exerted no effect on the mRNA expression of the aforementioned genes, relative to those in the surgery group **(** **Figure 1F** **)**. Moreover, the serum levels of IL-1β and IL-6 in the surgery group significantly increased relative to those in the sham group; by contrast, these serum levels were decreased with JWH133 treatment (**Figures 1G, H** **)**. Owing to the importance of IL-1β as an inflammatory factor influencing POCD (29, 31), we subsequently investigated the expression of IL-1β on astrocytes in the hippocampus. The surgical procedure markedly increased the IL-1β expression in the hippocampus relative to that in the sham mice; this effect on IL-1β expression was reversed with JWH133 treatment **(** **Figure 1I** **)**. Finally, we investigated changes in mouse behavior, which were analyzed using new object recognition and Y-maze tests. As shown in **(** **Figures 1J, K** **)**, in the novel object recognition test, the exploration time of a new object is significantly higher in the JWH133 mice than in the surgery mice. Using the same object across training trials, we observed no difference in the discrimination index between the inhibitor group and the surgery group. Y-maze tests showed similar results as those in the new object recognition examinations. The exploration time was longer in the surgery+JWH133 group in the novel arm than that in the surgery group in the Y maze experiment. These findings indicate that CBR2 activation was closely linked to the reduction in lipid accumulation and inflammation on astrocytes.

### Interference of CB2R on Astrocytes Exacerbated Lipid Accumulation in POCD

**Figure 2:**
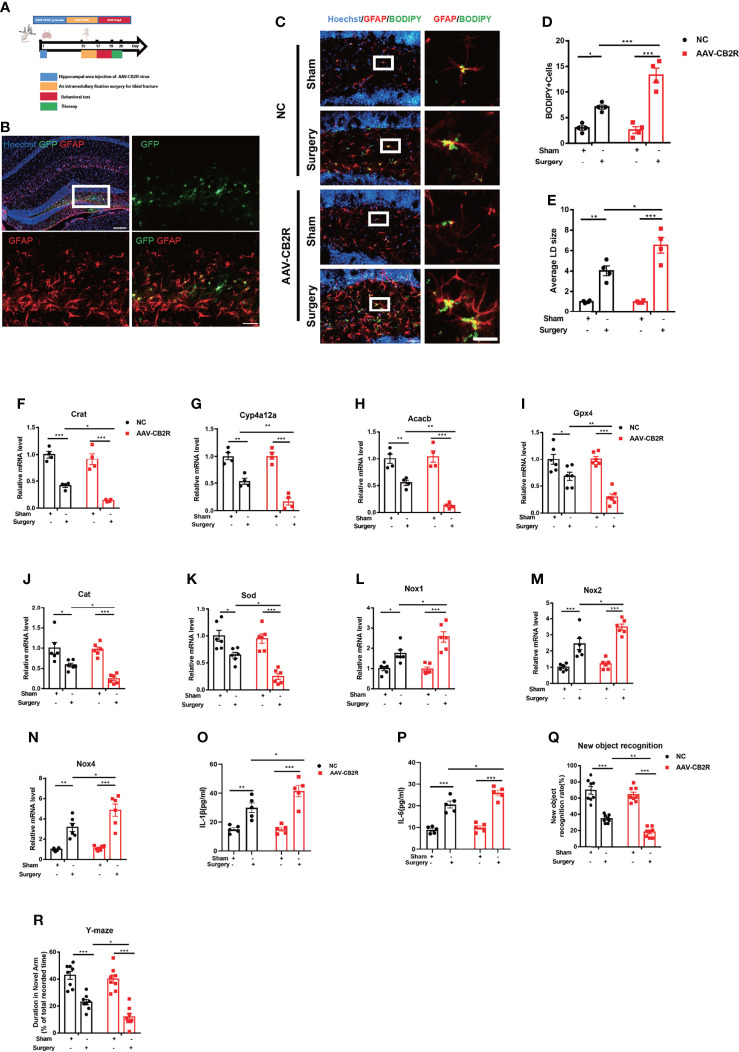
**(A)** Experimental protocol and timeline. Injection with the AAV virus in the hippocampus of mice 2 weeks before surgery; behavioral and biological testing 2 d after surgery. **(B)** Immunofluorescence staining of GFAP and AAV virus–GFP staining in the hippocampal DG brain region. **(C–E)** Immunofluorescence staining of GFAP and BODIPY staining in the hippocampal DG brain region; statistical data on BODIPY+ cells and the average LD size (means±SEM, n=4, scale bars=40 and 3 μm). **(F–N)** RT-qPCR results of the relative mRNA expression levels of Crat, Cyp4a12a, Acacb, Gpx4, Sod, Cat, and Nox1,2,4 in the hippocampus (means±SEM, Crat, Cyp4a12a, Acacb, n=4; Sod, Cat, and Nox1,2,4 n=6). **(O, P)** ELISA of serum IL-1β and IL-6 (n=5). **(Q, R)** New object recognition and Y-maze testing of mice in each group (means±SEM, n =10). ^*^ *P* < 0.05, ^**^ *P* < 0.01, ^***^ *P* < 0.001 compared with the corresponding group, as determined by two-way ANOVA, F (1, 12) _BODIPY Cells Interaction_=17.22; F (1, 12) _LD size Interaction_=7.598; F (1, 12) _Crat Interaction_=2.169;F (1, 12) _Cyp4a12a Interaction_=8.219; F (1, 12) _Acacb Interaction_=9.162;F (1, 20) _Gpx4 Interaction_=7.022;F (1, 20) _Cat Interaction_=3.355;F (1, 20) _Sod Interaction_=4.956;F (1, 20) _Nox1 Interaction_=6.025;F (1, 20) _Nox2 Interaction_=4.125;F (1, 20) _Nox4 Interaction_=4.494;F (1, 16)_IL-1βInteraction_=4.600;F (1, 16) _IL-6 Interaction_=3.384; F (1, 28) _New object recognition Interaction_=3.258;F (1, 28) _Y-maze Interaction_=2.318, followed by Tukey’s *post hoc* test.

To further define the role of CB2R on astrocytes in the POCD model, adeno-associated virus (AAVs) carrying the astrocyte-specific promoter GFAP were used to deliver siRNA to knock down CB2R on astrocytes **(** **Figures 2A, B** **)**. Conditional disruptions of CB2R exacerbated lipid accumulation after surgery, in contrast to that in the NC-Surgery group **(** **Figures 2C–E** **)**. RNA levels were further measured by real-time PCR to assay lipid metabolism-related genes and the antioxidant capacity in the hippocampus of the central brain area, relative to those in the NC-Surgery group. The expression levels of Crat, Cyp4a12a, Acacb **(** **Figures 2F–H** **)** Gpx4 (an antioxidant peroxidase), Sod, and Cat were significantly downregulated **(** **Figures 2I–K** **)** meanwhile, the expression of the NADPH oxidase subunit Nox1,2,4 was markedly upregulated with AAVs injection in the AAV-CB2R surgery group **(** **Figures 2L–N** **)**. Moreover, the serum levels of IL-1β and IL-6 in the AAV-CB2R surgery group significantly increased relative to that in the NC surgery group **(** **Figures 2O, P** **)**. Finally, behavioral testing, which included both the new-object-recognition and Y-maze tests, revealed that interfering with CB2R on astrocytes could markedly exacerbate cognitive impairment in mice. This effect was manifested in the reduction of the new object recognition index and the shortening of the retention time for the new arm **(** **Figures 2Q, R** **)**. Thus, CB2R on astrocytes affected lipid accumulation and inflammation in mice with POCD, and CB2R knockdown aggravated cognitive damage in POCD.

### CB2R Activation on Astrocytes Reduced Nerve Damage in POCD

**Figure 3:**
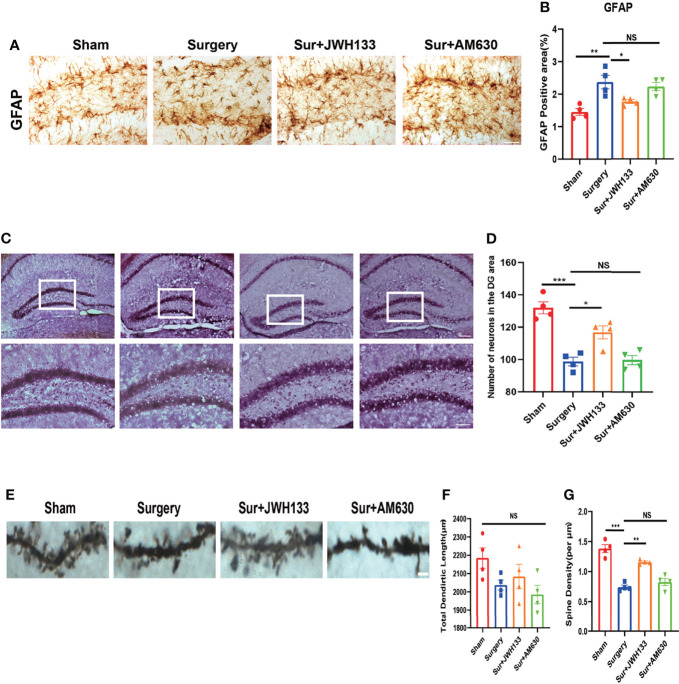
**(A, B)** GFAP expression analyzed by immunochemistry in the hippocampal dentate gyrus brain region; statistical data depicted in the graph (means±SEM, n=4, scale bars=100 μm). Quantification of positive areas by using ImageJ. **(C, D)** Nissl staining of the hippocampus in **(C)** and statistical graph is shown in **(D)** (n=4, scale bars =200 μm and 100μm). Examination of the hippocampus by Golgi–Cox staining **(E–G)**; pictures analyzed with the NeuroJ plugin in ImageJ to calculate the total length of dendrites and the density of synaptic spines (means±SEM, n=4, scale bars=4 μm). NS means not significant, *P < 0.05, **P < 0.01, ***P < 0.001 compared with the corresponding group, as determined by ANOVA.

To further explore whether CB2R exerted a neuroprotective effect on the hippocampus after activation, we did the following experiment. GFAP expression was measured to determine whether CB2R activation had an effect on glial activation induced by surgery and anesthesia. Significant glial cell activation was observed in the surgery group, but JWH133 administration significantly attenuated astrocyte activation **(** **Figures 3A, B** **)**. To verify this result, we performed Nissl staining in sections of the brain and observed a decrease in the number of neurons in the JWH133-treated surgery group; no such effects were exerted by the CB2R inhibitors, which was consistent with previous results **(** **Figures 3C, D** **)**. We subsequently assessed the neuronal synapses in the hippocampus. Golgi–Cox staining was used to examine the density of dendritic spines in the hippocampal region, which is considered highly associated with learning and memory (32). The spine density was lower in the surgery group than in the sham group. JWH133 treatment improved the decrease in surgery-induced spine density; no such reduction was observed in the AM630-treated group. However, no difference in total dendritic length was found between these groups **(** **Figures 3E–G** **).** Collectively, these results suggest that CB2R activation mitigated surgery-induced nerve damage.

### CB2R Activation Suppressed Lipopolysaccharide-Induced Lipid Drot Accumulation and Lipid Peroxidation, Which Mediated Mitochondrial Damage to U87 Cells

**Figure 4:**
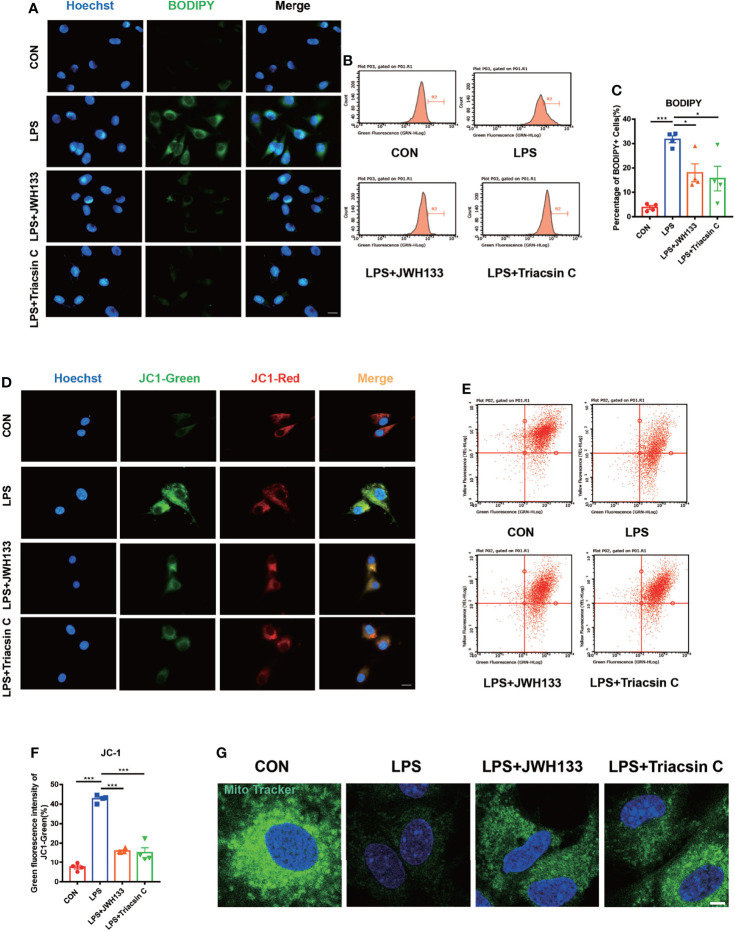
**(A)** U87 cells incubated with 1 μM of JWH133 or 1 μM of Triacsin C for 1 h and stimulated with 100 ng/mL of LPS for 6 h, collected for fluorescence photography (Scale bar= 20 μm) and cell flow analysis. **(B)** Statistical data in **(C)** (means±SEM, for 4 independent experiments). **(D)** JC1-green fluorescence and JC1-red fluorescence measured by flow cytometry in each group (scale bar=20 μm). **(E)** Flow cytometric analyses of the mitochondrial function by JC-1 assay and green fluorescence intensity of JC1-green. **(F)** Flow cytometry of the green fluorescence intensity of JC1-green (means±SEM, for 4 independent experiments). **(G)** After cell treatment, cells were incubated with Mitochondrial Tracker Green for 30 minutes at 37°C, and images were captured under confocal microscope (scale bar=50μm). NS means not significant, ^*^ *P* < 0.05, ^**^ *P* < 0.01, ^***^ *P* < 0.001 compared with the corresponding group, as determined by ANOVA.

Lipopolysaccharide (LPS)-induced LDs in BV2 cells were previously found to resemble those in microglia from aged mice (25). Recent studies have shown that LD accumulation increases lipid peroxidation-mediated stress and accelerates mitochondrial dysfunction. It also increases the expression of inflammatory factors (33–36). LPS has previously been used to simulate an *in vitro* model of POCD (37). In the current study, we used BODPIY to specifically stain the lipid molecules in U87 cells after LPS stimulation. LD deposition was observed after LPS treatment, which was reversed by treatment with the CB2R agonist JWH133. Lipid accumulation was also reversed in Triacsin C-treated U87 cells. Triacsin C inhibits long-chain acyl-CoA synthetase, which suppresses the *de novo* synthesis of glycerolipids and prevents lipid droplet formation (**Figure 4A**). We subsequently analyzed the green fluorescence of intracellular LDs by flow cytometry, which measured lipid levels. Consistent with previous research findings, JWH133 and Triacsin C attenuated LPS-induced lipid accumulation, and fluorescence intensity markedly decreased (**Figures 4B, C** **)**. Alterations in LDs can trigger dysfunction in many intracellular organelles, particularly mitochondria. Oxidative stress is an important risk factor for mitochondrial injury (38). We examined the mitochondrial membrane potential by using the JC-1 fluorescent dye and flow cytometry analysis and found that LPS induced a reduction in the membrane potential. However, compared with LPS administration, pre-administration of JWH133 significantly increased the membrane potential; however, this increase was reversed with the pre-administration of Triacsin C **(** **Figures 4D–F** **)**. Mitotracker Green was used to detect the number of mitochondria. The number of mitochondria was markedly decreased when LPS stimulated, treatment with JWH133 administration or lipid synthesis suppression significantly alleviated damage to the mitochondrial number **(** **Figures 4G** **)**, which was consistent with previous study findings.

### CB2R Activation Reduced TFEB-S211 Phosphorylation, Which Promoted Autophagy Levels

**Figure 5:**
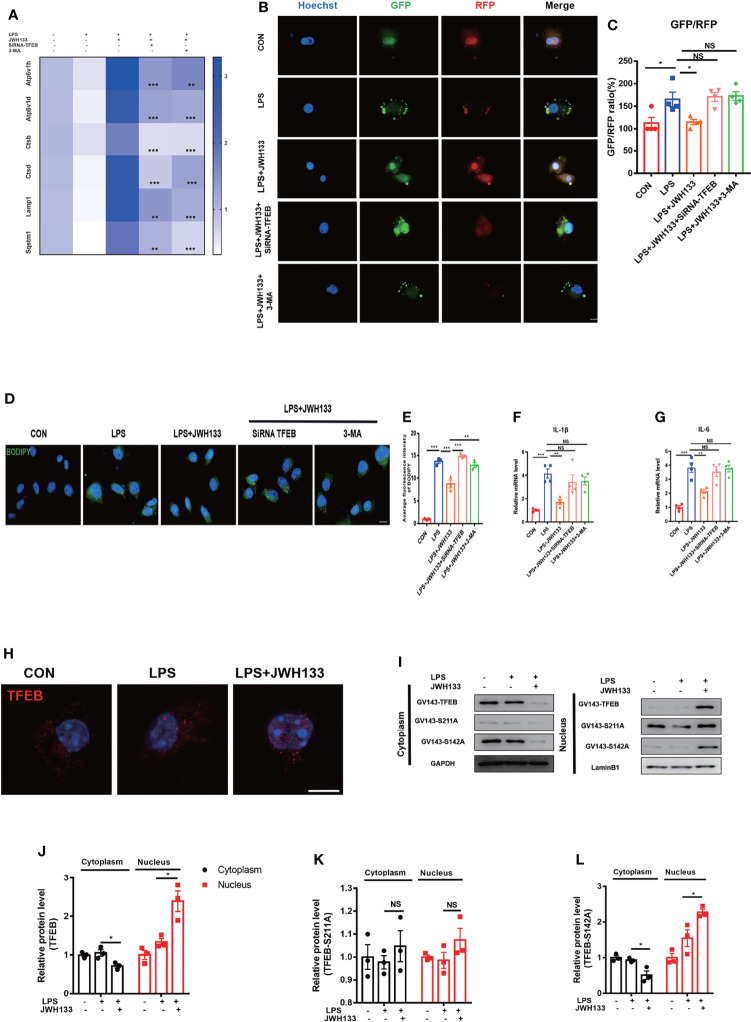
**(A)** U87 cells incubated with 5 mM of 3-MA and 1 μM of JWH133 for 1 h and then stimulated with 100 ng/mL of LPS for 6 h. U87 cells in the LPS+JWH133+SiRNA-TFEB group, mediated by SiRNA–TFEB for 48 h before treatment, collected for Q-PCR analysis (Sqstm1, Lamp1, Ctsd, Ctsb, Atp6v1d, Atp6v1h, and LPS+JWH133+SiRNA-TEEB LPS+JWH133+3-MA compared with LPS+JWH133; Statistical difference indicated in the figure; means±SEM, for 4 independent experiments). **(B, C)** U87 cells used in experiments 4 d after infection with the RFP-GFP-MAP1LC3B adenovirus. Cells treated using the same procedure as before. Determination of the relative GFP-to-RFP ratio (for 4 independent experiments, scale bar=20 μm). **(D, E)** BODIPY staining and statistical data for U87 cells (for 4 independent experiments, scale bar=20 μm). **(F, G)** Q-PCR analysis of IL-1β and IL-6 (means±SEM, for 4 independent experiments). **(H)** U87 cells tested for TFEB expression by using immunofluorescence (Scale bar=50 μm). **(I–L)** Transfection of GV143-TFEB, GV143-S211A, and GV143-S142A plasmids into U87 cells, cytoplasmic nucleoprotein extraction, and Western blot detection. Data shown in the figure (means±SEM, for 3 independent experiments). NS means not significant, ^*^ *P* < 0.05, ^**^ *P* < 0.01, ^***^ *P* < 0.001 compared with the corresponding group, as determined by two-way ANOVA, F (2,12) _TFEB Interaction_=22.83; F (2,12) _TFEB -S211A Interaction_= 0.05; F (2,12) _TFEB -S142A Interaction_= 24.81, followed by Tukey’s *post hoc* test.

LDs can generally be degraded by the autophagy-lysosomal pathway to minimize their intracellular accumulation. Autophagic dysfunction exacerbates pathological damage in multiple neurodegenerative diseases such as AD and PD (39–41). We previously reported that autophagy dysfunction was a major cause of CNS inflammation in POCD (21). The degradation of lipid droplets *via* autophagy is referred to as lipophagy (42). To determine whether CB2R was involved in lipophagy, we first assessed the mRNA expression levels of the autophagy–lysosomal pathway genes and found that LPS exposure led to the downregulation of autophagy-related genes—Sqstm1, Lamp1, Ctsd, Ctsb, Atp6v1d, and Atp6v1h. By contrast, with JWH133 treatment, these genes were upregulated relative to LPS **(** **Figure 5A** **)**. Previous literature reviews indicate that TFEB is a key regulator of autophagy and lysosome biogenesis. The phosphorylated form of TFEB is generally inactive and is sequestered in the cytoplasm. Meanwhile, TFEB is activated by dephosphorylation and translocated to the nucleus, where it is involved in the expression of autophagy–lysosomal pathway genes under starvation and stress conditions (43). Thus, we hypothesized that JWH133 upregulated autophagy-related genes *via* the key molecular TFEB. On the basis of the JWH133 treatment, we interfered with TFEB expression in U87 cells and found that the expected upregulation of autophagy-related genes was not realized in JWH133-related cells. Effect of JWH133 was also attenuated by the autophagic inhibitor 3-MA (**Figure 5A**). For a more intuitive visualization of cellular autophagy green fluorescent protein (GFP)-red fluorescent protein (RFP) MAP1LC3B double fluorescent adenovirus was transfected into U87 cells. In the acidic environment, the GFP fluorescence decreased with a reduction in pH, whereas RFP fluorescence exhibited stability. Autophagy flow is commonly measured by the two-point GFP-to-RFP ratio. In the present study, the green fluorescence decreased, whereas the red fluorescence increased when the cells were treated with JWH133. Conversely, the fluorescence changed in the opposite direction after co-treatment with 3-MA and interference with TFEB expression **(** **Figures 5B, C** **)**. The BODIPY staining of the cells also confirmed that interference with TFEB expression in U87 cells eliminated the effect of JWH133. This action was manifested by the enhancement of intracellular BODIPY fluorescence and an increase in lipid accumulation. Similarly, the autophagy inhibitor 3-MA improved lipid accumulation **(** **Figures 5D, E** **)**. In addition, pre-administration of JWH133 reduced the mRNA levels of IL-1β and IL-6 relative to those in the CON group; however, this decrease was countered by SiRNA-TFEB and 3-MA **(** **Figures 5F, G** **)**. We then performed immunofluorescence experiments, which revealed that JWH133 promoted TFEB translocation into the nucleus **(** **Figure 5H** **)**. In general, phosphorylated TFEB is sequestered in the cytoplasm, whereas dephosphorylated TFEB translocate into the nucleus and promotes the transcription of downstream genes. The activity and subcellular localization of TFEB are regulated by phosphorylation and dephosphorylation on residues S142 and S211. In our research, we found that in serine 142-to-alanine mutations, the nuclear translocation of TFEB was similar to that in the wild type. However, in the serine 211-to-alanine mutations, the retention of TFEB-S211A in the cytoplasm was not apparent **(** **Figures 5I-L** **)**. This observation confirms that JWH133 activated CB2R, reduced TFEB phosphorylation on Serine 211, and promoted TFEB into the nucleus. These results clearly indicate that the intracellular LDs were ultimately degraded by autophagy *via* TFEB.

### CB2R Activation Promoted TFEB Binding to PGC1α and Regulated Lipid Metabolism

**Figure 6:**
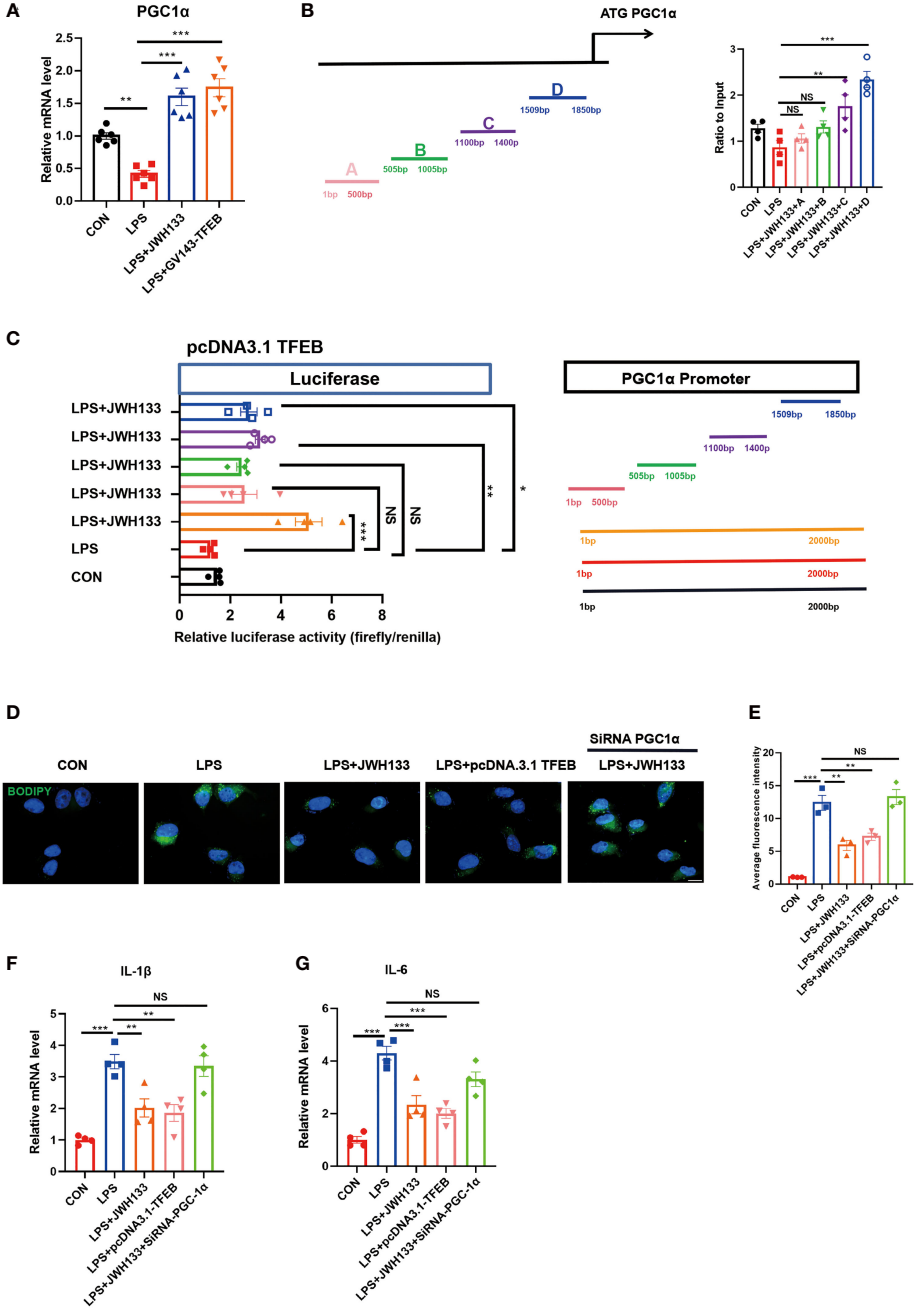
Transfection of GV143-TFEB plasmid into U87 cells. **(A)** After treatment of cells with LPS and JWH133, mRNA is extracted from U87 cells and PGC-1 expression is detected by Q-PCR (means±SEM, for 6 independent experiments). **(B)** ChIP analysis of U87 cells. (means±SEM, for 4 independent experiments). **(C)** Representative diagrams of constructs containing the PGC1α promoter region with either the intact or truncated promoter region of PGC1α. Luciferase activity measured after transfecting increasing amounts of TFEB-Flag combined with PGC1α plasmids (means±SEM, for 4 independent experiments). **(D, E)** U87 cells transfected with the TFEB-flag plasmid or mediated by PGC1α expression, processed with LPS and JWH133, and stained with BODIPY. Data shown in the figure. (means±SEM, for 3 independent experiments). **(F, G)** Q-PCR analysis of IL-1β and IL-6 (means±SEM, for 4 independent experiments). NS means not significant, *P < 0.05, **P < 0.01, ***P < 0.001 compared with the corresponding group, as determined by ANOVA.

To explore functional studies between TFEB and lipid metabolism. Together with previous findings, we found TFEB controls lipid metabolism, inflammation by inducing autophagy, and regulating PGC1α expression (17). In the current study, we intended to confirm whether TFEB directly regulated PGC1α expression; mRNA was extracted from U87 cells, and statistical data showed that CB2R activation promoted PGC1α expression, whereas interference with TFEB eliminated this effect **(** **Figure 6A** **)**. To determine whether PGC1α was a direct target of TFEB, we analyzed its promoter and identified possible binding sites. The possible binding sites of TFEB and PGC1α were predicted using the JASPAR (https://jaspar.genereg.net/) and UNIPROT (https://beta.uniprot.org/) databases. ChIP-qPCR of U87 cells showed that TFEB could bind two site of the PGC1α promoter regions **(** **Figure 6B** **)**. Further, Luciferase reporter assay showed that LPS decreased the luciferase activity of PGC1α luciferase reporters obviously. However, administration of JWH133 increased the activity of luciferase activity compared with the LPS group. Subsequently, the PGC1α promoter region was cut in sections and tested for luciferase activity. We found that the 1-500bp and 505-1005bp sites of the promoter did not increase the transcriptional activity of PGC1α, while the two regions of 1100-1400bp and 1509-1850bp could significantly increase the activity of luciferase (**Figure 6C** **)**. BODIPY staining results showed that TFEB overexpression in U87 cells could reduce lipid accumulation **(** **Figures 6D, E** **)**, apart from reducing the expression levels of inflammatory factors IL-1β and IL-6 in the cells, as determined by Q-PCR analysis. After interference with PGC1α expression, the effects of JWH133 and pcDNA3.1 TFEB were also canceled **(** **Figures 6F, G** **)**. These results indicate that when CB2R was activated, TFEB directly regulated PGC1α gene expression.

## Discussion

**Figure 7:**
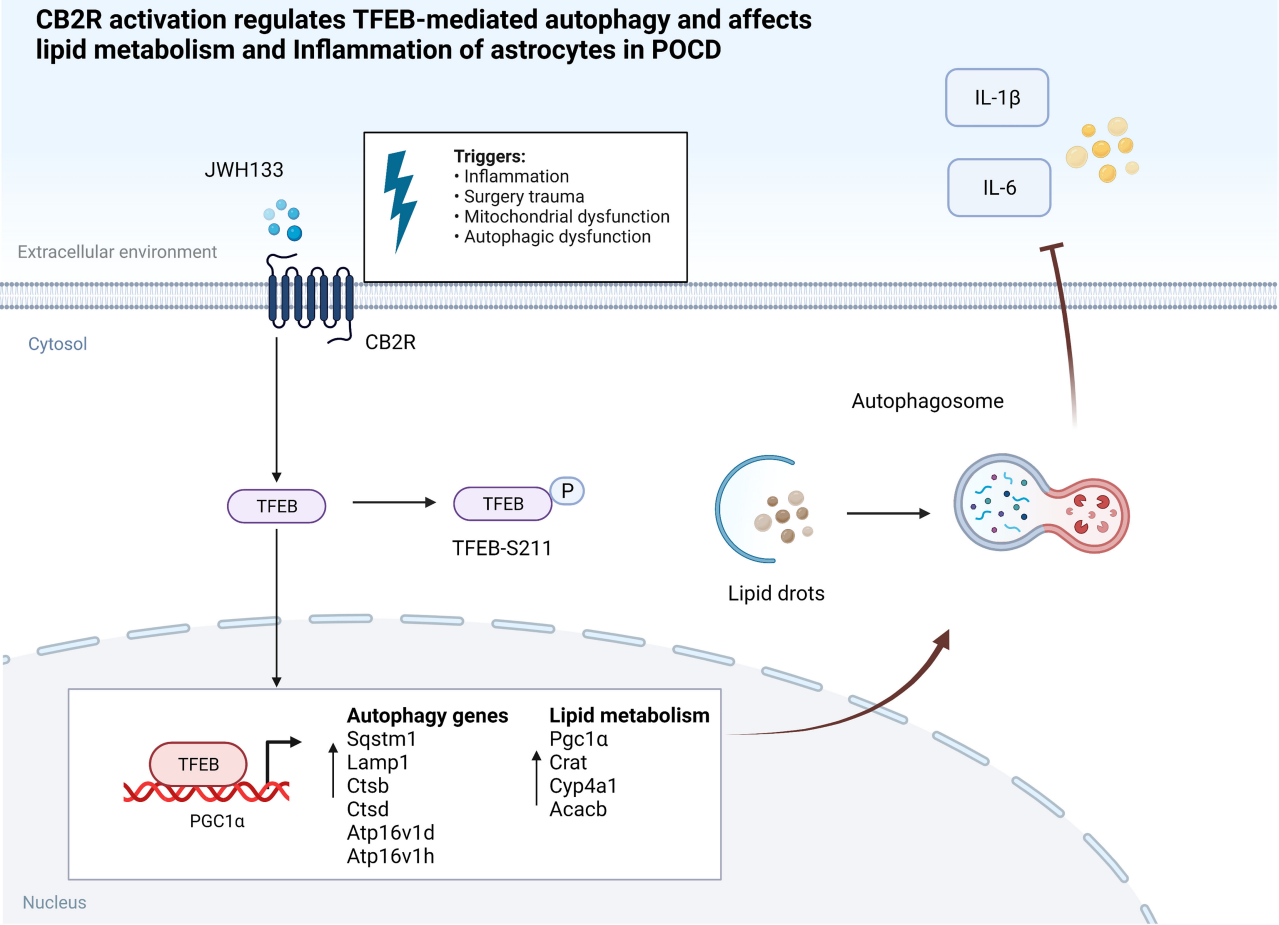
CB2R activation regulates TFEB-mediated autophagy and affects lipid metabolism and inflammation of astrocytes in POCD.

In the current study, we found that CB2R activation regulated lipid metabolism and inflammation *via* the autophagic pathway and improved cognitive performance in mice with POCD. Astrocyte-specific promoter viruses were then injected into the hippocampal brain region of mice. The accumulation of astrocyte lipids in the brains of mice with POCD was exacerbated, and impairment of the postoperative learning memory was subsequently aggravated. Notably, interference with TFEB expression in U87 cells abolished the protective effect of JWH133 and impeded PGC1α transcription. This influenced the expression of downstream lipid metabolism genes and reduced the accumulation of lipids and inflammatory factors in U87 cells. JWH133 reduced the phosphorylation at the TFEB S211 site, thus promoting TFEB nucleation and autophagy. Further, TFEB binding to the PGC1α promoter region contributed to PGC1α transcription. The mechanism allowing the CB2R modulation of lipid metabolism is illustrated in **Figure 7**. The findings of this study suggest that it can be a potential new therapeutic target for POCD.

POCD is a common complication in elderly patients who undergo surgery involving anesthesia (44). Despite numerous studies on the mechanisms of POCD, the underlying pathogenesis remains inconclusive. Recent studies suggest that glial cell dysfunction can potentially contribute to the development and progression of POCD (45, 46). Astrocytes, which are the most abundant glial cells in the CNS, play a primary role in synaptic transmission and information processing in neural circuits, which participate in neuroinflammation and multiple processes in CNS disorders (47). Activated mast cells can trigger astrocyte activation and thus lead to POCD (48). Another set of data suggests that astrocyte-derived chemokine C-C motif ligand 2 mediates microglial activation is also a potential therapeutic target for POCD (49). In our research, the mechanism of CB2 activation in astrocytes was explored *in vitro* by using the U87 astrocyte cell line. Unlike neurons, astrocytes engage in lipid metabolism when lipids that are isolated in the cytoplasm generate cytotoxicity, and astrocytes can release antioxidants and effectively manage oxidative stress (50). The lipids metabolized during dissociation are typically toxic and impair the integrity of the mitochondrial membrane (51). A large volume of evidence indicates that age-related neurodegenerative diseases are mostly associated with lipid metabolism and inflammation in astrocytes (25, 52–55). Autophagy activation in our previous study reduced POCD in mice (21). In the present study, we found that CB2R activation reduced lipid accumulation and increased the expression of genes related to lipid metabolism while reducing the expression of inflammatory factors. Specific interference with CB2R on astrocytes exacerbated lipid accumulation and the release of inflammatory factors IL-1β and IL-6. Moreover, CB2R agonists altered lipid accumulation and inflammatory factors in astrocytes in postoperative mice. Together, these results demonstrated that the activation of the CB2R effectively reduced cognitive impairment in POCD. Thus, astrocyte response is crucial in the pathological progression of POCD.

As a GPCR, CB1R usually triggers psychiatric disorders as a side effect, which impedes its application as a therapeutic target (56). Meanwhile, CB2R produces less severe psychiatric-like side effects in the brain, particularly in neurodegenerative diseases, neurodevelopment (57). Emerging evidence suggests that modulation of the cannabinoid system exerts neuroprotective effects in various neurologic disorders (58). Treatment with the CB2R agonist JWH133 suppresses postsurgical neuroinflammation and enhances memory, whereas treatment with the CB2R antagonist AM630 exacerbates neuroinflammation and worsens postsurgical memory (11). In the present study, JWH133 decreased astrocyte activation, neuronal loss, and lipid accumulation in astrocytes in mice with POCD. It also alleviated damage to prominent neural spines and improved cognitive ability in mice. Exposure of U87 cells to JWH133 led to a significant reduction in LPS-induced lipid accumulation and reduced mitochondrial damage *via* the autophagic pathway. This pathway was blocked by the autophagy inhibitor 3-MA and the Triacsin C long-chain fatty acyl coenzyme A synthase inhibitor.

Notably, we found that TFEB-S211 dephosphorylation promoted the nuclear entry of TFEB, which regulates the expression of autophagy-related genes (59). In U87 cells, inhibiting TFEB expression also abolished the effect of JWH133 and increased autophagy blockade. Notably, PGC1α is the direct target molecule of TFEB, which binds to the promoter region of PGC1α. The construction of a truncated promoter region revealed that CB2R activation promoted TFEB binding to the 1100-1400bp and 1509-1850bp sites. Simultaneously, TFEB plasmid overexpression reduced lipid accumulation caused by LPS in U87 cells. After PGC1α interference, the protective effect of JWH133 was canceled, and lipid accumulation was aggravated in U87 cells. These results also verified coordinated autophagy induction and PGC1α-mediated lipid catabolism and inflammation *via* TFEB to control nerve damage induced by surgery and anesthesia. Therefore, our study clarified a new therapeutic target for CB2R-mediated postoperative cognitive dysfunction.

## Data Availability Statement

The original contributions presented in the study are included in the article/Supplementary Materials. Further inquiries can be directed to the corresponding authors.

## Ethics Statement

All experimental procedures were carried out in accordance with the Chinese Guidelines of Animal Care and Welfare, the animal study was reviewed and approved by Medical Animal Experiment Center of Nanchang University.

## Author Contributions

FH and GX conceived and designed the experiments. LZ, XW, WY, JY, XF, DY, and YF performed the experiments and analyzed the data. PF, QZ, JH, SC, and GW managed the literature searches and figure drawing. LZ and FH wrote the manuscript. GX, XW, and WY revised the manuscript. FX, YL, and XL proofread the language. All authors have read and approved the final manuscript.

## Funding

This work was supported by the National Natural Science Foundation of China (82060219, 81760261 and 81860259), the Province Natureal Science Foundation of Jiangxi(20212ACB216009; 20202BAB206033 and 20212BAB216048), “Thousand Talents Program” of Jiangxi Province (JXSQ2019201023), Youth Team Project of the Second Affiliated Hospital of Nanchang University (2019YNTD12003), The Provincial Education Foundation of Jiangxi (GJJ190094), and Health Commission Foundation of Jiangxi Province (20204361 and 20171075).

## Conflict of Interest

The authors declare that the research was conducted in the absence of any commercial or financial relationships that could be construed as a potential conflict of interest.

## Publisher’s Note

All claims expressed in this article are solely those of the authors and do not necessarily represent those of their affiliated organizations, or those of the publisher, the editors and the reviewers. Any product that may be evaluated in this article, or claim that may be made by its manufacturer, is not guaranteed or endorsed by the publisher.

## Abbreviations

POCD, Postoperative cognitive dysfunction; LDs, lipid droplets; CB2R, Cannabinoid receptor type II; FA, fatty acids; CB1R, cannabinoid receptor type I; ICV, Intracerebroventricular; AD, Alzheimer’s disease; PD, Parkinson’s disease; TBI, and traumatic brain injury; Gpx4, glutathione peroxidase 4; NOX2, NADPH oxidase 2; SOD, Superoxide dismutase; Nox1, NADPH Oxidase 1; Nox4, NADPH Oxidase 4; CAT, Catalase; MAP1LC3B, microtubule-associated protein 1 light chain 3 beta; SQSTM1, sequestosome 1; 3-MA, 3-methyladenine; ATG5, autophagy protein5; AAV, adeno-associated virus.
